# HIV-1 Transmissions Among Recently Infected Individuals in Southwest China are Predominantly Derived from Circulating Local Strains

**DOI:** 10.1038/s41598-018-29201-3

**Published:** 2018-08-27

**Authors:** Jianjun Li, Yi Feng, Zhiyong Shen, Yingxin Li, Zhenzhu Tang, Runsong Xiong, Hongman Zhang, Jing Wei, Xinjuan Zhou, Yueqin Deng, Ningye Fang, Guanghua Lan, Shujia Liang, Qiuying Zhu, Hui Xing, Yuhua Ruan, Yiming Shao

**Affiliations:** 1 Institute of HIV/AIDS Prevention and Control, Guangxi Zhuang Autonomous Region Center for Disease Control and Prevention, Nanning, China; 2 State Key Laboratory of Infectious Disease Prevention and Control (SKLID), National Center for AIDS/STD Control (NCAIDS) and Prevention, Chinese Center for Disease Control and Prevention (China CDC), Collaborative Innovation Center for Diagnosis and Treatment of Infectious Diseases, Beijing, China; 3 College of Pharmacy, Guangxi Medical University, Nanning, China

## Abstract

Although the Guangxi region accounts for 10% of all HIV-1 cases new reported in 2011 in China, the sources of the transmitted HIV-1 strains are virtually unknown. To determine the extent to which recent HIV infections were derived from already circulating local strains as opposed to recently introduced strains, we performed a cross-sectional molecular epidemiological investigation of recent infections across Guangxi during 2012–2013. HIV-1 nucleotide sequences were amplified and sequenced. Phylogenetic analyses of *pol* gene regions were used to determine HIV-1 transmission source strains. Based on 229 sequences generated, the subtype/CRF distribution was as follows: CRF01_AE (61.1%), CRF07_BC (18.8%), CRF08_BC (16.6%), CRF55_01B (3.1%), and subtype B′ (0.4%). In total, 213 of 229 (93.0%) sequenced transmission strains were derived from already-circulating local strains. Multivariate logistic regression analysis showed that only an age of 18–25 years was significantly associated with transmission from outside Guangxi (compared to >25 years, AOR: 5.15, 95% CI: 1.18–22.48, *p* < 0.01). This is the first study to use a Bayesian discrete phylogeographic approach to analyze transmission source strains in China. Our results provide useful data for designing evidence-based prevention strategies and methods for combating the rapid spread of sexually transmitted HIV in Guangxi.

## Introduction

The Guangxi Zhuang Autonomous Region (Guangxi) is located in southwest China. Due to its location along a major heroin trafficking route linking Guangxi with Yunnan and Vietnam and its close proximity to the world’s major heroin-producing area, known as the Golden Triangle, human immunodeficiency virus (HIV) transmission in Guangxi was primarily fueled initially by intravenous drug use^1^. In 1996, the first case of HIV infection among Guangxi local residents was identified in an intravenous drug user (IDU) in Pingxiang city, close to Vietnam^2^. HIV prevalence among IDUs has increased rapidly since then^3^. HIV infection through drug injection accounted for 69% of reported HIV cases in Guangxi in 2003, but the proportion of HIV infections transmitted through sexual intercourse increased to 66% in 2009^3,4^. Among the 31 provinces and municipalities in China, Guangxi has one of the highest rates of reported HIV infection, accounting for approximately 10% of reported HIV cases in 2011 in China^4^. Since the end of 2011, heterosexual transmission (90%) has been the predominant transmission route^4^. Because heterosexual transmission is the primary method by which HIV spreads from high-risk groups^5^ to the general population, the HIV epidemic poses greater challenges than ever before in Guangxi.

In 1996, four HIV subtypes were identified in Guangxi, consisting of subtypes B′ (Thai B), C, D, and CRF01_AE^2^. Subtypes C and CRF01_AE were being transmitted via IDUs and heterosexual transmission, whereas subtypes B′ and D were circulating among commercial blood donors. Subsequent sequence analysis showed two geographically distinct, highly homogeneous HIV-1 strains in Guangxi^6,7^. B/C intersubtype recombinants were found in IDUs from Baise, near the Yunnan-Guangxi border. Circulating recombinant-form (CRF) AE strains (now referred to as CRF01_AE) were found in IDUs from Pingxiang, near the China-Vietnam border, and from Nanning, the capital city of Guangxi^7^. Although CRF07_BC and CRF08_BC were first detected among IDUs in Guangxi in 1997^7,8^, it is likely that these two CRFs were initially established in Yunnan Province^9,10^ and spread through overland heroin trafficking routes to Guangxi and Xinjiang^1^. Sequence analysis showed that CRF07_BC and CRF08_BC were closely related and may have evolved from a common parental strain^11^. CRF01_AE, originated in central Africa in the 1970s and spread in Thailand in the 1980s through heterosexual transmission^12,13^, first identified among IDUs in Guangxi, showed significant clustering with strains found in northern Vietnam^6,14^. Subsequently, a national molecular epidemiological survey conducted across China in 2006 showed that there were three major HIV-1 subtypes circulating in Guangxi, i.e., CRF01_AE, CRF08_BC, and CRF07_BC, accounting for 60.0%, 29.1%, and 6.4% of cases, respectively^15^. Other studies performed from 2008 to 2009 showed that CRF01_AE was the dominant subtype in Guangxi^16,17^. Two large CRF01_AE clusters were identified among heterosexual transmission cases in Guangxi^18,19^. One cluster originated from Vietnamese strains that were previously reported in IDUs. A second, novel cluster was also identified and showed a close relationship to strains from the Fujian province of China.

HIV-1 molecular epidemiological surveys of recent infections are important for understanding the real-time dynamics of the HIV-1 epidemic in Guangxi and the complexities of HIV-1 subtype transmission. The BED-capture enzyme immunoassay (BED-CEIA) has been widely used to measure the proportion of HIV-1-specific IgGs among total IgGs in blood samples for the purpose of identifying recent infections^20,21^. However, BED-CEIA classifies some individuals having long-standing infections as recently infected^22,23^. Use of a combination of non-serological biomarkers, such as CD4 cell counts, with BED-CEIA offers an alternative method for the purpose of accurately identifying recent infections^24,25^. In this study, we performed a cross-sectional molecular epidemiological survey among recently infected individuals identified by a combination of BED-CEIA and CD4 results. We further used bioinformatics approaches to analyze the sources of HIV-1 transmission strains in Guangxi, China.

## Results

### Demographic and epidemiological characteristics of study participants

Among 275 plasma samples from individuals recently infected with HIV-1 from 2012 to 2013, *pol* sequences were successfully amplified and genotyped from a total of 229 samples (83.3%). The demographic and epidemiological characteristics of the 229 genotyped individuals are summarized in Table 1. Among the study participants, 64.6% were males, and the mean age of the participants was 44.9 ± 16.1 years. In total, 66.8% (153/229) of participants were of Han ethnicity, 27.1% (62/229) were of Zhuang nationality, and 6.1% (14/229) were of other minority ethnicities, consisting of Yao, Mulao, Tujia, and Dong. The median CD4 cell count was 515 cells/µl (IQR: 419–604). Individuals were classified into the following risk groups: heterosexual (202/229, 88.2%), MSM (18/229, 7.9%), IDUs (4/229, 1.7%), and unknown (5/229, 2.2%). In terms of the distribution of cases among geographic regions, 71 cases were from central Guangxi (31.0%), consisting of 48 cases from Nanning, 15 from Guigang, and eight from Laibin; 46 cases were from southwestern Guangxi (20.1%), consisting of 24 from Qinzhou, eight from Baise, seven from Beihai, six from Chongzuo, and one from Fangchenggang; 60 cases were from northwestern Guangxi (26.2%), consisting of 26 from Guilin, 26 from Liuzhou, and eight from Hechi; and 52 cases were from southeastern Guangxi (22.7%), consisting of 22 from Yulin, 16 from Hezhou, and 14 from Wuzhou.

**Table 1:** Demographic and epidemiological characteristics and distribution of HIV-1 genotypes among recently infected individuals in Guangxi, China.

Characteristics		Total	HIV-1 Genotypes									
			CRF01_AE		CRF07_BC		CRF08_BC		CRF55_01B		B	
			No.	%	No.	%	No.	%	No.	%	No.	%
Total		229	140	61.1	43	18.8	38	16.6	7	3.1	1	0.4
Sex	Male	148	85	57.4	36	24.3	21	14.2	6	4.1	0	0.0
	Female	81	55	67.9	7	8.6	17	21.0	1	1.2	1	1.2
Age (years)	18–25	33	19	57.6	7	21.2	2	6.1	4	12.1	1	3.0
	26–49	101	60	59.4	20	19.8	18	17.8	3	3.0	0	0.0
	≥50	95	61	64.2	16	16.8	18	18.9	0	0.0	0	0.0
Ethnicity	Han	153	95	62.1	31	20.3	23	15.0	3	2.0	1	0.7
	Zhuang	62	37	59.7	11	17.7	11	17.7	3	4.8	0	0.0
	Other^a^	14	8	57.1	1	7.1	4	28.6	1	7.1	0	0.0
Marital Status	Married	132	82	62.1	22	16.7	24	18.2	3	2.3	1	0.8
	Unmarried	62	32	51.6	17	27.4	9	14.5	4	6.5	0	0.0
	Divorced/Widowed	35	26	74.3	4	11.4	5	14.3	0	0.0	0	0.0
Region^b^	Central	71	37	52.1	17	23.9	15	21.1	2	2.8	0	0.0
	Southwestern	46	25	54.3	8	17.4	13	28.3	0	0.0	0	0.0
	Northwestern	60	43	71.7	9	15.0	5	8.3	3	5.0	0	0.0
	Southeastern	52	35	67.3	9	17.3	5	9.6	2	3.8	1	1.9
CD4 (cells/µl)	350–500	104	59	56.7	23	22.1	20	19.2	2	1.9	0	0.0
	501–1000	118	78	66.1	18	15.3	17	14.4	4	3.4	1	0.8
	>1000	7	3	42.9	2	28.6	1	14.3	1	14.3	0	0.0
Risk group^c^	Hetero	202	126	62.4	34	16.8	36	17.8	5	2.5	1	0.5
	MSM	18	7	38.9	8	44.4	1	5.6	2	11.1	0	0.0
	IDU	4	3	75.0	0	0.0	1	25.0	0	0.0	0	0.0
	Unknown	5	4	80.0	1	20.0	0	0.0	0	0.0	0	0.0

^a^Other minority ethnicities, consisting of Yao, Mulao, Tujia, and Dong.

^b^Central, consisting of Nanning, Guigang, and Laibin; northwestern, consisting of Guilin, Liuzhou, and Hechi; southeastern, consisting of Yulin, Hezhou, and Wuzhou; southwestern, consisting of Qinzhou, Baise, Beihai, Chongzuo, and Fangchenggang.

^c^Hetero, heterosexual; MSM, men who have sex with men; IDU, intravenous drug user.

### HIV-1 genotypes and clusters

**Figure 1:**
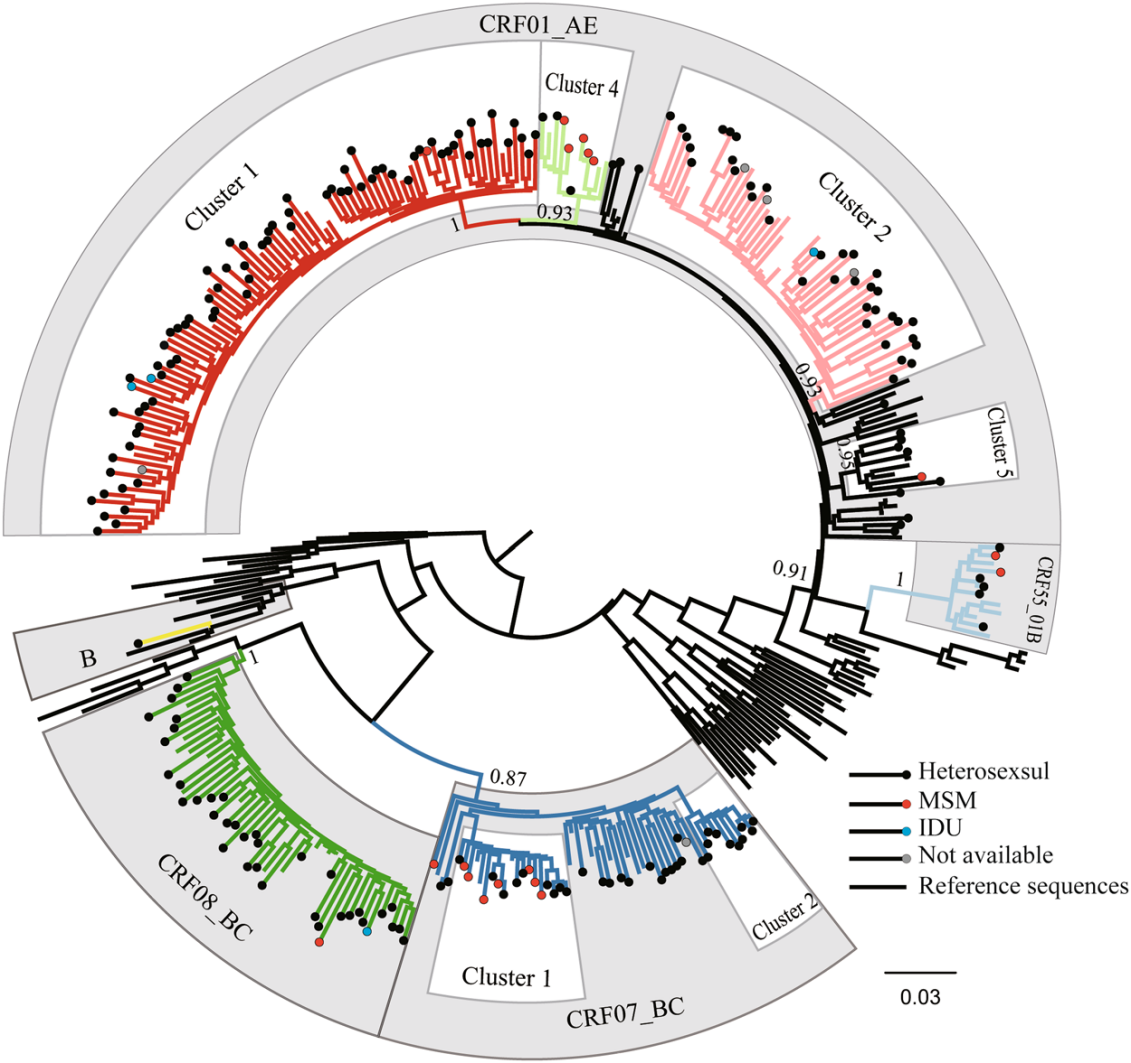
Maximum-likelihood phylogenetic trees of HIV-1 pol sequences from 229 recently infected individuals in Guangxi, China. The stability of each node was assessed by bootstrap analyses with 1000 replicates, and the bootstrap values were shown at the corresponding nodes. The branches with dots at the ends represent sequences of study participants, and the others without dots represent references.

**Figure 2:**
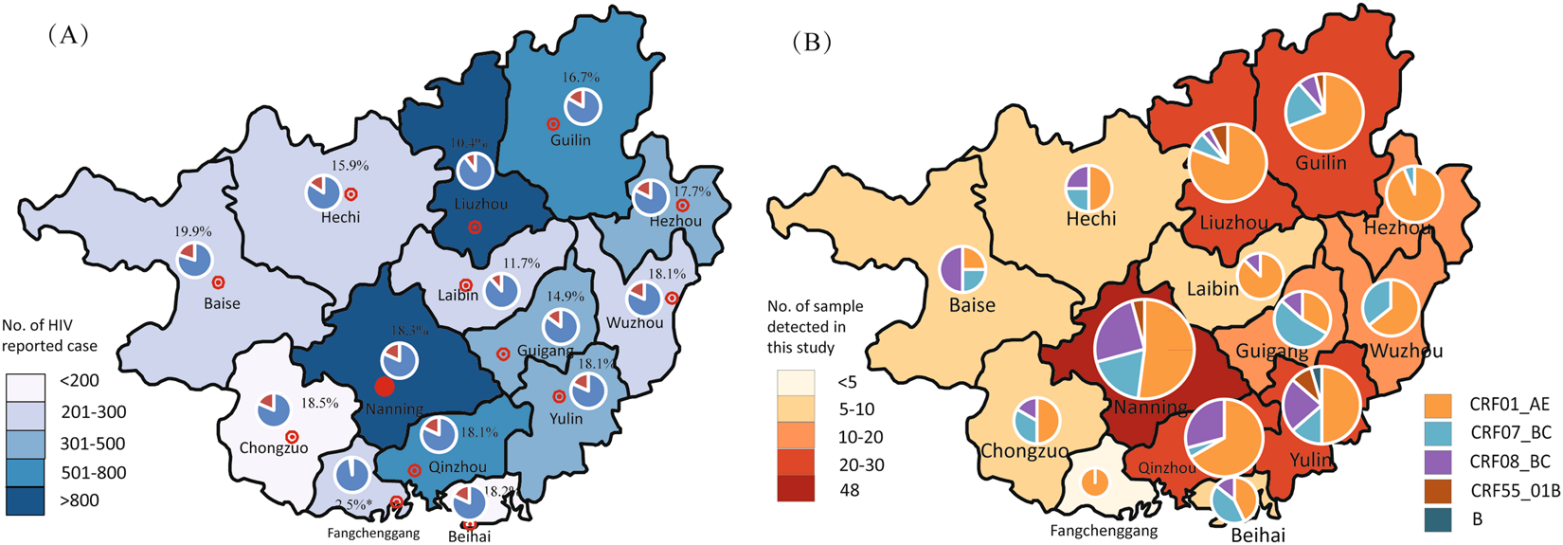
(**A**). Number of reported cases and proportion of recently infections in different prefectures in Guangxi from July 2012 and June 2013. (Illustrated based on the dataset tabulated in Table S1). (**B**) Distribution of HIV-1 genotypes of recently infections in each prefecture across Guangxi (Illustrated based on the dataset tabulated in Table 1).

Among the 229 *pol* sequences, 140 (61.1%), 43 (18.8%), 38 (16.6%), seven (3.1%), and one (0.4%) were identified as CRF01_AE, CRF07_BC, CRF08_BC, CRF55_01B, and subtype B, respectively (Fig. 1, Table 1). Distribution of HIV-1 genotypes of recently infections in each prefecture were shown in Fig. 2(B). The greatest diversity of HIV subtypes and CRFs was observed among the heterosexual population, with CRF01_AE, CRF07_BC, CRF08_BC, CRF55_01B, and subtype B′ accounting for 62.4% (126/202), 16.8% (34/202), 17.8% (36/202), 2.5% (5/202), and 0.5% (1/202) of cases, respectively. Among the MSM population, CRF01_AE, CRF07_BC, CRF08_BC, and CRF55_01B accounted for 38.9% (7/18), 44.4% (8/18), 5.6% (1/18), and 11.1% (2/18) of cases, respectively. The HIV-1 genotype distributions of the heterosexual (n = 202) and MSM (n = 18) populations were significantly different (Fisher’s exact test, χ^2^=12.582, *p* = 0.009). Among the IDU population, CRF01_AE and CRF08_BC accounted for 75.0% (3/4) and 25.0% (1/4) of cases, respectively. The genotypes CRF01_AE (80.0%, 4/5) and CRF07_BC (20.0%, 1/5) were found in the five individuals whose risk status was unknown.

As shown in Fig. 1, among 140 CRF01_AE sequences, 131 (93.6%) were grouped into four clusters: CRF01_AE Cluster 1 (77/140, 55.0%), CRF01_AE Cluster 2 (41/140, 29.3%), CRF01_AE Cluster 4 (7/140, 5.0%), and CRF01_AE Cluster 5 (6/140, 4.3%). A further nine (6.4%) sequences remained ungrouped (Fig. 1). CRF01_AE Cluster 1 strains were found among 73 heterosexuals, one MSM, two IDUs, and one individual of unknown risk group. CRF01_AE Cluster 2 strains were found among 37 heterosexuals, one IDU, and three individuals of unknown risk group. CRF01_AE Cluster 4 strains were found among three heterosexuals and five MSMs. CRF01_AE Cluster 5 strains were found among five heterosexuals and one MSM. Among 43 CRF07_BC sequences, two clusters were identified among 26 (60.5%) sequences, consisting of CRF07_BC Cluster 1 (16/43, 37.2%) and CRF07_BC Cluster 2 (10/43, 23.3%), whereas 17 (39.5%) sequences were not grouped (Fig. 1). CRF07_BC Cluster 1 strains were found among nine heterosexuals and seven MSMs, and CRF07_BC Cluster 2 strains were found among 10 heterosexuals. All seven CRF55_01B sequences (100%) clustered together into a single cluster of five heterosexuals and two MSMs. Among 38 CRF08_BC sequences, no clusters were identified (Fig. 1).

### Sources of HIV-1 transmission strains

**Figure 3:**
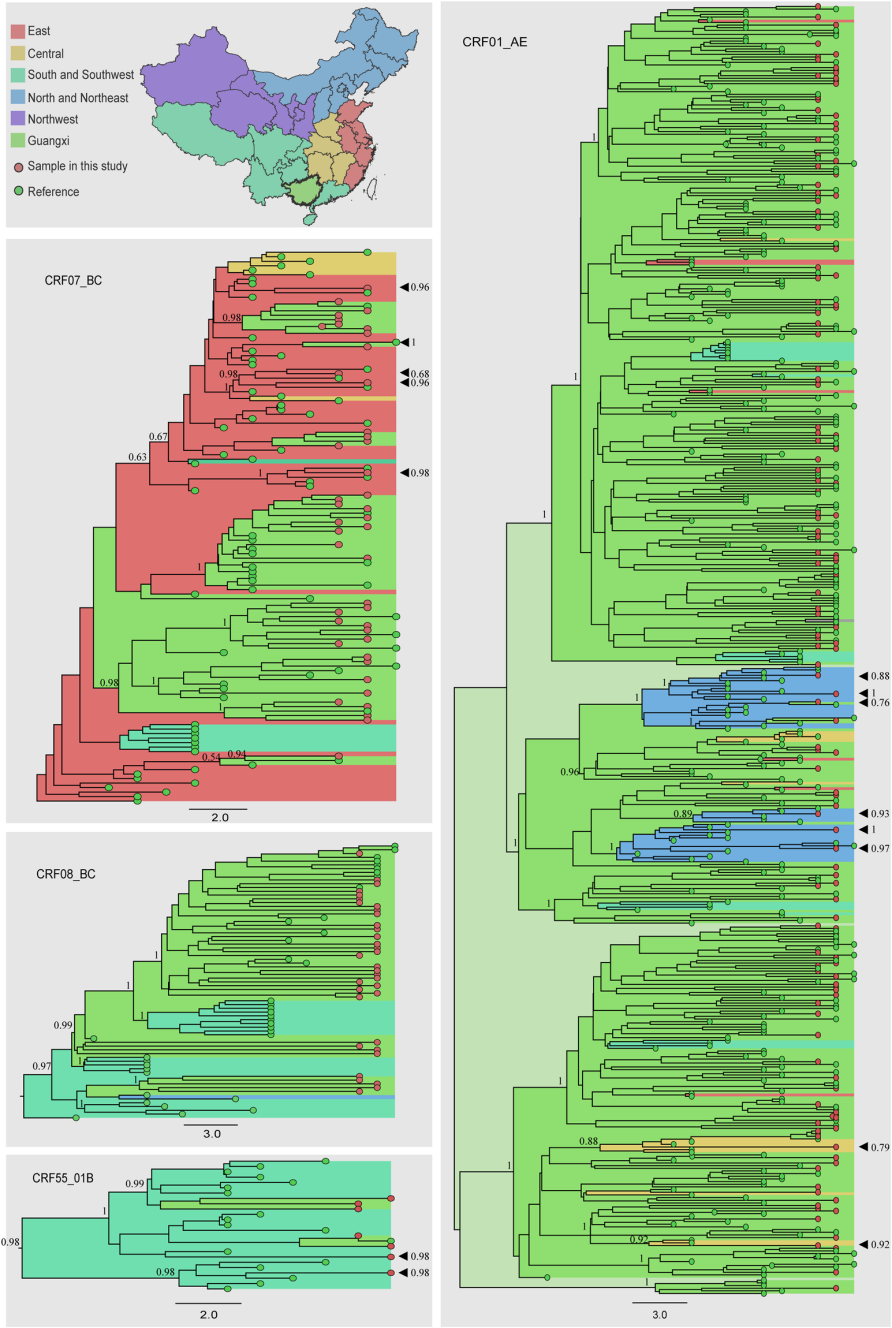
Time-scaled phylogeographic history of recent infections in Guangxi. Background colors around branches represent the most probable origin of the parental node of each branch. Respective colors for each region are shown in the sketch map of China in the upper left, which was drawn using Adobe Illustrator CS6. The values of probability inferred by Bayesian skygrid demographic model estimation for the most probable ancestral location were shown at the corresponding nodes. Red and green dots at the ends of branches represent sequences of study participants and references, respectively. Study participants with sequences not derived from Guangxi are indicated with black triangles.

The sources of HIV-1 transmission strains among recently infected individuals in Guangxi were estimated by reconstruction of Bayesian discrete phylogeographic approaches under a Bayesian skygrid demographic model (See Method). The results of estimation were visualized on maximum clade credibility (MCC) trees (Fig. 3) and summarized in Table 2. Among 229 *pol* gene sequences, 213 (93.0%) were derived from strains already circulating in Guangxi, with the remaining 16 (7.0%) derived from strains circulating outside Guangxi. As shown in Table 2, sources of HIV transmission differed by sex, age, marital status, and risk group of the study participants. The proportion of source strains derived from those circulating outside Guangxi was 2.5% among female and 9.5% among male participants (*p* = 0.047); 21.2% among participants aged 18–25 years, 5.9% among those aged 26–49 years, and 3.2% among those aged ≥50 years (*p* = 0.002); 3.0% among married, 14.5 among unmarried, and 8.6% among divorced/widowed participants (*p* = 0.013); and 5.9% among heterosexual and 22.2% among MSM participants (*p* = 0.024). Multivariate logistic regression analysis showed that only age was significantly associated with a source of HIV-1 transmission from outside of Guangxi, with those aged 18–25 years being at highest risk (compared to >25 years, AOR: 5.15, 95% CI: 1.18–22.48, *p* < 0.01). Among a total of 16 HIV strains found to have been introduced from outside Guangxi, the most probable origins were Beijing (five strains), Shanghai (five strains) and Shenzhen (two strains) (Fig. 3). The most probable origins of the remaining four strains were four different provinces, none of which were the neighboring provinces of Yunnan, Guizhou, Hunan, or Guangdong.

**Table 2:** Analysis of HIV-1 transmission strain sources among recently infected individuals in Guangxi, China.

Characteristics		No. of cases	Strains circulating in Guangxi		Strains circulating outside Guangxi		χ^2^	*p*
			No.	%	No.	%		
Total		229	213	93.0	16	7.0		
Sex	Female	81	79	97.5	2	2.5		
	Male	148	134	90.5	14	9.5	3.936	0.047
Age (years)	18–25	33	26	78.8	7	21.2		
	26–49	101	95	94.1	6	5.9		
	≥50	95	92	96.8	3	3.2	12.589	0.002
Ethnicity^a^	Han	153	142	92.8	11	7.2		
	Other	76	71	93.4	5	6.6	0.029	0.864
Marital Status	Married	132	128	97.0	4	3.0		
	Unmarried	62	53	85.5	9	14.5		
	Divorced/widowed	35	32	91.4	3	8.6	8.723	0.013
Region^b^	CSR	117	111	94.9	6	5.1		
	NSR	112	102	91.1	10	8.9	1.272	0.259
Risk Group^c^	Hetero	202	190	94.1	12	5.9		
	MSM	18	14	77.8	4	22.2		
	Other	9	9	100	0	0	7.445	0.024

^a^Other minority ethnicities, consisting of Zhuang, Yao, Mulao, Tujia, and Dong.

^b^CSR, central and southwestern region, consisting of Nanning, Guigang, Laibin, Qinzhou, Baise, Beihai, Chongzuo, and Fangchenggang; NSR, northwestern and southeastern region, consisting of Guilin, Liuzhou, Hechi, Yulin, Hezhou, and Wuzhou.

^c^Hetero, heterosexual; MSM, men who have sex with men; Other, intravenous drug users and unknown.

## Discussion

Guangxi is a relatively poor area in rural southwestern China that accounts for approximately 19% of new reported HIV cases in 2011 in China. Although traditional field epidemiological surveys focusing on HIV infection and related risk factors have been conducted, this is the first study to use molecular epidemiology to track the sources of HIV transmission strains among recently infected individuals in China. In this study, we found that 93.0% of new HIV-1 infections were derived from strains already circulating in Guangxi. In a study of the international metropolis Shanghai, the transmission network was found to include not only strains circulating in Shanghai itself but also those transmitted between Shanghai and other provinces^26^. The most probable sources of HIV transmission strains circulating outside Guangxi in our study were from the large Chinese cities of Beijing, Shanghai, and Shenzhen, which illustrates the complexities of large sexual networks arising from population mobility in China. Young individuals in Guangxi were more likely to have been infected with an HIV strain circulating outside Guangxi, indicating increased mobility and higher-risk sexual activities in this population group.

Changes in HIV epidemic characteristics and new trends among recent HIV infections in Guangxi were also identified in this study. In total, five HIV-1 subtypes and CRFs, CRF01_AE, CRF07_BC, CRF08_BC, CRF55_01B, and subtype B′, were detected among study participants in Guangxi. CRF01_AE, CRF07_BC, and CRF08_BC are the three major HIV-1 genotypes circulating in China, whereas CRF55_01B and subtype B′ are minor HIV-1 genotypes. Recent studies have reported that CRF01_AE is the dominant subtype in Guangxi^15,17,27^, which is consistent with the findings of our study. However, the proportion of CRF01_AE (61.1%) cases in this study was lower than that previously found. Earlier studies found that the proportion of CRF01_AE cases was 77.3% among 481 treatment-naïve HIV-1-infected participants in 2009–2010^17^ and 83.1% among 260 participants who were newly diagnosed, treatment naïve, and 16–25 years of age in the cities of Liuzhou, Hezhou, and Nanning in Guangxi in 2009–2013^27^. As previously reported by Feng *et al*.^28^, HIV-1 CRF01_AE strains circulating in China can be categorized into seven independent lineages. In this study, four of these lineages were identified among CRF01_AE strains circulating in Guangxi, corresponding to the CRF01_AE Clusters 1, 2, 4, and 5 identified by Feng *et al*.; no strains from CRF01_AE Clusters 3, 6, or 7 were found in this study. Among the four CRF01_AE clusters identified in this study, CRF01_AE Cluster 1 accounted for more than half of the total, which is consistent with the findings of some recent studies^27,28^. This indicates that CRF01_AE Cluster 1 strains are the major strains circulating in Guangxi. CRF01_AE Cluster 2 strains, which originated among early IDU populations in Guangxi^7^ and sexually transmitted populations from northern Vietnam^29^, accounted for one-third of the total. We also found that CRF01_AE Clusters 1 and 2 predominated among heterosexuals, whereas CRF 01_AE Cluster 4 predominated among MSMs, which is consistent with the findings of a previous study^28^.

The proportion of CRF07_BC (18.8%) strains in this study was higher than that found in previous studies, in which the proportions of CRF07_BC were 6.4% among 110 HIV-positive persons newly diagnosed in 2006^15^ and 7.3% among 481 treatment-naïve HIV-1-infected participants in 2009–2010 in Guangxi^17^. In addition to CRF07_BC Cluster 1, which corresponds to the cluster identified by Li *et al*.^30^, a novel CRF07_BC Cluster 2, composed of ten sequences, was identified in this study. The proportion of CRF08_BC appears to vary across studies, from 29.1% in a 2006 study^15^, to 11.4% in a 2009–2010 study^17^, to 16.6% in this study. Moreover, no clusters were identified among CRF08_BC strains. These findings indicate that CRF07_BC may be experiencing an increasing epidemic trend, whereas infection with CRF08_BC has decreased among recently infected individuals in Guangxi. It is possible that the new wave of HIV infections among heterosexuals and MSMs is being rapidly driven by subtype CRF07_BC virus.

CRF55_01B, a novel HIV-1 CRF composed of CRF01_AE and subtype B with four recombination breakpoints in the *pol* gene, was first identified from three epidemiologically unlinked MSMs in China^31^. CRF55_01B was found among seven recently infected individuals in our study: five heterosexuals and two MSMs. Although CRF55_01B has been circulating widely among MSMs in major cities in southern, eastern, and central China^32^, the spread of HIV-1 CRF55_01B among both heterosexuals and MSMs may foster future HIV epidemics in Guangxi.

The majority of strains we detected in Guangxi in recently infected individuals were CRF01_AE, CRF07_BC, CRF08_BC and CRF55_01B. Only one subtype B′ strain was detected. All of these strains have been found in China for many years. Circulating recombinant forms such as CRF07_BC, CRF08_BC, and CRF55_01B even originated in China^2,5,24^. Moreover, the CRF01_AE strain in China originated from Thailand and Vietnam through early cross-border transmission in the 1990s^25^. However, several clusters of CRF01_AE that are unique to China have been characterized, which indicates that strains of CRF01_AE have been localized in China^25^. At least two CRF01_AE clusters transmitted mainly through heterosexuals and IDUs have been characterized in Guangxi^24^. In this study, our results indicate that the epidemic of HIV in Guangxi is mainly driven by local transmission. Although some strains from other provinces of China are present, we did not find any evidence to support cross-border transmission in recently infected individuals.

Our study has several limitations. A sampling bias might be present if there are many HIV-infected individuals in Guangxi whose infections have not been detected and diagnosed. Bias may also have been introduced in our representative HIV-1 *pol* gene reference sequence dataset, as not all individuals living with HIV have had their virus sequenced and not all sequences have been submitted to GenBank. Additionally, our method of determining HIV-1 transmission strain sources may have some limitations. We were not able to determine the direct source of each transmission, as intermediaries may exist between a case and its transmission source. However, our method does identify infections that are closely linked.

In summary, our study found that HIV-1 transmission in Guangxi mainly occurs from strains already circulating locally. We also found a high diversity of HIV-1 strains and recombinants co-circulating among recently infected individuals in Guangxi. These results may aid in designing evidence-based prevention strategies and methods for countering the rapid spread of this sexually transmitted epidemic in Guangxi. Future studies should focus on using HIV transmission networks to inform real-time prevention and intervention strategies.

## Methods

### Study participants and blood collection

Eligible study participants included newly diagnosed HIV-positive cases 18 years or older having a positive BED-CEIA test and a first CD4 cell count >350 cells/μl within three months of western blotting (WB) diagnosis. Exclusion criteria for study participants included AIDS, antiretroviral therapy (ART), and a first CD4 cell count <200 cells/μl within three months of WB diagnosis^22,23^. In total, 6,647 HIV/AIDS cases aged ≥18 years were newly diagnosed between July 2012 and June 2013 in 14 prefectures in Guangxi, China. Excluding those ineligible for the BED-CEIA test, 3,739 plasma samples, which were also diagnosed as HIV positive by western bolting (WB), were shipped to our Laboratory of HIV/AIDS Confirmation and stored at −80 °C for subsequent tests. All of these samples were tested for recent infection through the BED-CEIA test^22,23^, and 896 individuals were identified as positive. Among them, 608 individuals had first CD4 cell counts >350 cells/μl within three months of WB diagnosis and were defined as recently infected^22,23^. Among them, 275 plasma samples were randomly selected from 550 plasma samples having sufficient plasma volume and used for genotyping and phylogenetic analysis (For detailed sampling information, see Fig. 2(A), Supplementary Table S1 and Fig. S1). Socio-demographic data, containing sex, age, ethnicity, marital status, date of sampling, prefecture of sampling, date of HIV-positive diagnosis, transmission route, and first CD4 cell count within three months of WB diagnosis, were collected from the Guangxi HIV/AIDS case reporting system database at the Guangxi Zhuang Autonomous Region Center for Disease Control and Prevention.

### Ethics Statement

All HIV tests were informed and voluntary. Written consent for HIV testing was obtained, in which the participants agreed that if they were diagnosed with HIV infection, their plasma samples could be used in research for the purpose of controlling and preventing HIV. The study protocol was approved by the Ethics Review Committee of Guangxi Center for Disease Control and Prevention. All participants provided written informed consent. All samples of the participants were tested in this study as approved by Guangxi institutional review board (IRB) and anonymized for our study. This study and all methods were approved by the IRB of the Guangxi Center for Disease Control and Prevention. All research methods in this study were performed in accordance with the approved guidelines.

### HIV-1 RNA extraction, amplification, and sequencing

In total, 275 plasma samples identified as recently infected were used for genotype analysis. Viral RNA was extracted from 200 µl of plasma using the NucliSENS easyMAG platform (BioMérieux, Boxtel, Netherlands) according to the manufacturer’s instructions. Then, viral RNA was subjected to nested polymerase chain reaction (PCR) to obtain fragments of the *pol* gene (HXB2, positions 2147–3462 for a total of 1315 bp)^33^. The *pol* fragment was first amplified using One Step Reverse Transcription PCR (Takara, Dalian, China) with primers MAW26 (5′-TGGAAATGTGGAAAGGAAGGAC-3′ and RT21 (5′-CTGTATTTCTGCTATTAAGTCTTTTGATGGG-3′) in 25 µl reaction volumes. Cycling conditions were as follows: 50 °C for 30 min; 94 °C for 2 min; 30 cycles of 94 °C for 30 s, 55 °C for 30 s, and 72 °C for 2 min 30 s; and 72 °C for 10 min. Then, the second PCR was performed using 2 × Taq PCR MasterMix (Tiangen, Beijing, China) with primers Pro-1 (5′-CAGAGCCAACAGCCCCACCA-3′) and RT-20 (5′-CTGCCAGTTCTAGCTCTGCTTC-3′) in 50 µl reaction volumes. Cycling conditions were as follows: 94 °C for 5 min; 30 cycles of 94 °C for 30 s, 63 °C for 30 s, and 72 °C for 2 min 30 s; and 72 °C for 10 min. Each step was performed with appropriate negative controls to detect possible contamination during the experiments. PCR products were analyzed using 1% agarose gel electrophoresis. Positive PCR products were purified using QIAquick Gel Extraction Kit (Qiagen, Valencia, CA, USA) and sequenced directly on an ABI 3730XL automated sequencer using BigDye terminators (Applied Biosystems, Foster City, CA, USA) by Beijing Biomed Technology Development Co., Ltd (Beijing, China).

### HIV-1 phylogenetic analysis

HIV-1 genotypes were determined based on neighbor-joining tree analysis in comparison with Los Alamos 2013 HIV-1 subtyping references. The nucleotide sequences of 229 HIV-1 *pol* genes (PR-RT region) were aligned separately using Gene Cutter (http://www.hiv.lanl.gov/cotent/sequence/.html) with HIV-1 group M subtype reference sequences [Los Alamos National Laboratory (LANL) HIV Sequence Database (http://www.hiv.lanl.gov/cotent/sequence/NEWALIGN/align.html, accessed in April 2013)] as follows: A1 (3 sequences), A2 (3), B (4), C (4), D (4), F1 (4), F2 (4), G (4), H (4), J (3), K (2), CRF01_AE (3), CRF02_AG (3), CRF 06_cpx (3), CRF07_BC (3), CRF08_BC (2), and group N (3). In addition to these 56 reference strains, we used 74 reference sequences from viruses that represent subtypes/CRFs commonly identified in China as follows: CRF01_AE (37), CRF08_BC (11), CRF07_BC (9), CRF55_01B (5), CRF59_01B (5), CRF67_01B (2), CRF68_01B (3), and subtype B (2). We also used two CRF01_AE strains from the Central African Republic and four CRF01_AE strains from Vietnam for a total of 136 reference sequences. Following alignment, manual adjustments were made using BioEdit software^34,35^, taking into consideration protein coding sequences. Maximum-likelihood trees were generated in RAxML using a GTRGAMMA model. Bootstrap trees were produced using the rapid bootstrapping algorithm and 1000 bootstrap replicates^5^. Clusters with bootstrap value higher than 0.85 (85%) were defined as phylogenetic cluster.

### HIV-1 transmission strain source analysis

To determine the origins of the strains in recently infected individuals, all available *pol* gene (PR-RT region) sequences from China were downloaded from the Los Alamos HIV Sequence Database (8,607 sequences, accessed March, 2017). In addition, considering that Guangxi is located in southern China, sequences from neighboring countries and regions in South Asia were also downloaded (613 from Vietnam, 1,420 from Thailand, 240 from Myanmar, 424 from Malaysia, 265 from Philippines, 1,450 from Cambodia, 292 from Hong Kong and 443 from Taiwan). All the downloaded sequences were combined with the 8,536 PR-RT region sequences obtained from the Chinese national HIV surveillance and drug resistance monitoring database (not open access) to yield a total dataset of 22,290 sequences. A local BLAST database was built with all of the 22,290 sequences using the application *makeblastdb* within the BLAST+ software package. BLAST+ searches were performed for the 229 *pol* sequences of our study participants against the local BLAST database, and the five sequences most similar to each query were selected^36^. After removal of duplicate sequences, 707 *pol* sequences were used for determining HIV-1 transmission strain sources, consisting of 478 unique *pol* sequences identified by BLAST and 229 *pol* gene sequences from our study participants.

For more precise analysis and better display of phylogenetic trees, we separated the 707 *pol* sequences into four data sets based on genotype (CRF01_AE, CRF07_BC, CRF08_BC, or CRF55_01B), for datasets of 482, 127, 73, and 25 sequences, respectively (Supplementary Fig. S2). As there was only one sequence belonging to subtype B′, no phylogenetic tree was needed to determine transmission source. To reconstruct the spatial dynamics and estimate the sources of the strains of our study participants, a Bayesian discrete phylogeographic approach was performed using Markov chain Monte Carlo (MCMC) runs of 300 million generations with BEAST v.1.8.2 under a Bayesian skygrid demographic model. The first 10–30% of the states from each run were discarded as burn-in^37,38^. All four data sets were analyzed using a general time-reversible (GTR) model specifying a gamma distribution as a prior on each relative substitution rate and a relaxed uncorrelated lognormal (UCLN) molecular clock model to infer the timescale of HIV evolution with a gamma distribution prior on the mean clock rate (shape = 0.001, scale = 1000)^39^. The Bayesian MCMC output was analyzed using Tracer v1.6 (http://beast.bio.ed.ac.uk/Tracer). The most probable origin of the study participants was estimated according to output of the posterior of Bayesian estimation and visualized on maximum clade credibility (MCC) trees using the program FigTree v1.4.0 (http://beast.bio.ed.ac.uk) with the same calculation.

### Nucleotide sequence accession numbers

All HIV-1 *pol* gene nucleotide sequences obtained in this study were submitted to GenBank under accession numbers KY226003-KY226231.

### Statistical analysis

Statistical analyses for this study were performed using the SPSS 17.0 statistical analysis software package (SPSS Inc. Chicago, IL, USA). Categorical variables were compared using the chi-squared test. A stepwise multivariate logistic regression model was constructed to select the variables that were independently associated with HIV transmission strain sources. All tests were two-tailed, and *p* < 0.05 was considered statistically significant.

## Electronic supplementary material

Supplementary Information
