# Author Correction: HIV-1 Transmissions Among Recently Infected Individuals in Southwest China are Predominantly Derived from Circulating Local Strains

**DOI:** 10.1038/s41598-018-36020-z

**Published:** 2018-11-27

**Authors:** Jianjun Li, Yi Feng, Zhiyong Shen, Yingxin Li, Zhenzhu Tang, Runsong Xiong, Hongman Zhang, Jing Wei, Xinjuan Zhou, Yueqin Deng, Ningye Fang, Guanghua Lan, Shujia Liang, Qiuying Zhu, Hui Xing, Yuhua Ruan, Yiming Shao

**Affiliations:** 1 Institute of HIV/AIDS Prevention and Control, Guangxi Zhuang Autonomous Region Center for Disease Control and Prevention, Nanning, China; 2 State Key Laboratory of Infectious Disease Prevention and Control (SKLID), National Center for AIDS/STD Control (NCAIDS) and Prevention, Chinese Center for Disease Control and Prevention (China CDC), Collaborative Innovation Center for Diagnosis and Treatment of Infectious Diseases, Beijing, China; 3 College of Pharmacy, Guangxi Medical University, Nanning, China

Correction to: *Scientific Reports* 10.1038/s41598-018-29201-3, published online 27 August 2018

The Acknowledgements section in this Article is incomplete.

“The authors would like to thank Lingjie Liao (China CDC), and Xingguang Li (China CDC) for technical support and analysis of the data. The authors would also like to thank the Group for the HIV Molecular Epidemiologic Survey. Contributing members of the Group [name of contributor (facility of the contributor)]: Yi Luan (Nanning CDC); Houjin Ma (Guilin CDC); Xing Liu (Liuzhou CDC); Mei Li (Qinzhou CDC); Ruiguo Ye (Yulin CDC); Daquan Luo (Baise CDC); Yanfei Meng (Guigang CDC); Shuyin zhao (Hezhou CDC); Bingjian Liang (Wuzhou CDC); Tianji Huang (Chongzuo CDC); Baohui Tang (Hechi CDC); Shuzhi Chen (Laibin CDC). Hao Zhu (Beihai CDC), Weifei Pang (Fangchenggang CDC). We would like to thank Editage [www.editage.cn] for English language editing.”

should read:

“The authors would like to thank Lingjie Liao (China CDC), and Xingguang Li (China CDC) for technical support and analysis of the data. The authors would also like to thank the Group for the HIV Molecular Epidemiologic Survey. Contributing members of the Group [name of contributor (facility of the contributor)]: Yi Luan (Nanning CDC); Houjin Ma (Guilin CDC); Xing Liu (Liuzhou CDC); Mei Li (Qinzhou CDC); Ruiguo Ye (Yulin CDC); Daquan Luo (Baise CDC); Yanfei Meng (Guigang CDC); Shuyin zhao (Hezhou CDC); Bingjian Liang (Wuzhou CDC); Tianji Huang (Chongzuo CDC); Baohui Tang (Hechi CDC); Shuzhi Chen (Laibin CDC). Hao Zhu (Beihai CDC), Weifei Pang (Fangchenggang CDC). We would like to thank Editage [www.editage.cn] for English language editing.

This study was supported by the Guangxi Science and Technology Development plan (1298003-1-4), National Natural Science Foundation of China (grants 81360442, 81502862, 81460510), Guangxi Science and Technology Bureau (AB16380213), Guangxi Bagui Honor Scholars, Ministry of Science and Technology of China (grant 2012ZX10001-002), Chinese State Key Laboratory of Infectious Disease Prevention and Control, and the International Development Research Center of Canada (grant 104519-010).”

