# Supplementary material for: HIV-1 Transmissions Among Recently Infected Individuals in Southwest China are Predominantly Derived from Circulating Local Strains
Source: Sci Rep. 2018 Aug 27;8:12831. doi: 10.1038/s41598-018-29201-3 (PMC6110827; doi:10.1038/s41598-018-29201-3)
Supplement: Supplementary file 1 — Supplementary Information [file 41598_2018_29201_MOESM1_ESM.pdf]

Supplementary

HIV-1 Transmissions Among Recently Infected Individuals in Southwest China are Predominantly Derived from Circulating Local Strains

Jianjun Li[1¶], Yi Feng[2¶], Zhiyong Shen[1¶], Yingxin Li[3¶], Zhenzhu Tang[1*], Runsong Xiong[1], Hongman Zhang[1], Jing Wei[2], Xinjuan Zhou[1], Yueqin Deng[1],

Ningye Fang[1], Guanghua Lan[1], Shujia Liang[1], Qiuying Zhu[1], Hui Xing[2], Yuhua Ruan[2], Yiming Shao[2]

1 Institute of HIV/AIDS Prevention and Control, Guangxi Zhuang Autonomous Region Center for Disease Control and Prevention, Nanning, China
2 State Key Laboratory of Infectious Disease Prevention and Control (SKLID), National Center for AIDS/STD Control (NCAIDS) and Prevention, Chinese Center for Disease Control and Prevention (China CDC), Collaborative Innovation Center for Diagnosis and Treatment of Infectious Diseases, Beijing, China
3 College of Pharmacy, Guangxi Medical University, Nanning, China.

Table S1. Sample screening of recently infected cases for phylogenetic analysis of the study participants

Region	Prefecture	No. of newly reported HIV/AIDS cases (a)	No. of BED-CEIA test (b)	No. of HIV-1 recent infections by BED-CEIA (c)	No. of BED-CEIA positive and CD4 greater than 350 (d)	Ratio of recently infection (d/b)	No. of enough plasma volume among CD4 more than 350 cells/μl	No. of sampling for sequencing (e)	Sampling ratio (e/d)
Total		6647	3739	896	608	16.30%	550	275	45.23%
Central	Nanning	1519	920	278	168	18.30%	127	61	36.31%
	Guigang	481	249	58	37	14.90%	37	18	48.65%
	Laibin	266	146	26	17	11.70%	17	8	47.06%
Northwestern	Guilin	722	442	100	74	16.70%	71	35	47.30%
	Liuzhou	921	530	74	55	10.40%	55	30	54.55%
	Hechi	239	145	28	23	15.90%	17	8	34.78%
Southeastern	Yulin	473	259	67	47	18.10%	47	24	51.06%
	Hezhou	327	169	40	30	17.70%	30	16	53.33%
	Wuzhou	258	150	32	27	18.10%	27	14	51.85%
Southwestern	Qinzhou	585	325	93	59	18.10%	54	27	45.76%
	Baise	276	180	40	36	19.90%	36	18	50.00%
	Chongzuo	189	97	29	18	18.50%	17	8	44.44%
	Beihai	131	88	28	16	18.20%	14	7	43.75%
	Fangchenggang*	260	40	3	1	2.50%	1	1	100.00%

*** Among 260 newly reported HIV/AIDS cases in Fangchenggang city, excluded 121 cases ineligible for BED-CEIA test criterion and 99 cases plasma samples not shipped to BED-CEIA test,40 cases were tested by BED-CEIA.**

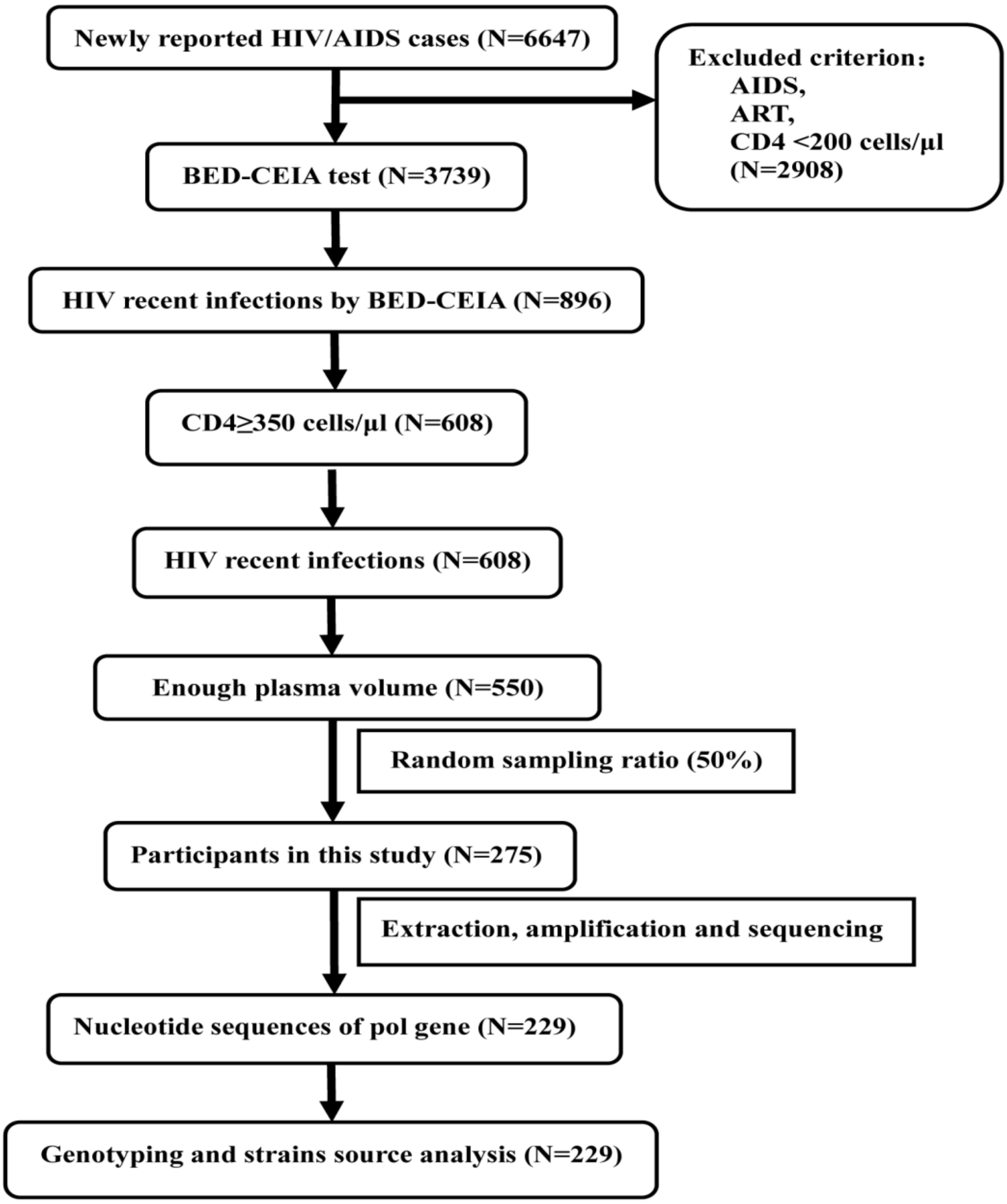

Fig S1. Flow chart of study profile. HIV detection assays and numbers of study samples are shown. ART, antiretroviral therapy; BED-CEIA, BED-capture enzyme immunoassay.

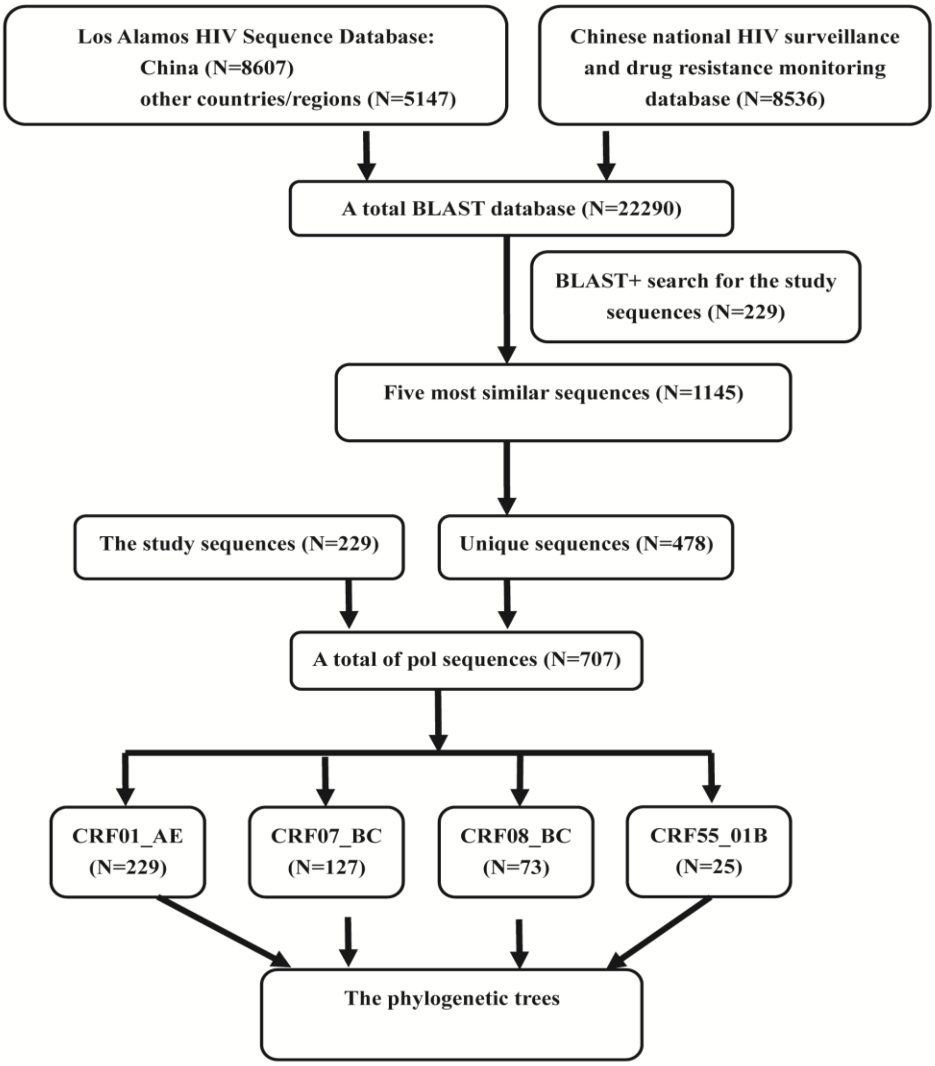

Fig S2. Flow chart of HIV-1 transmission strain source analysis. Numbers of sequences are shown in parentheses. BLAST, Basic Local Alignment Search Tool.
